# CT showed multiple pulmonary balloon lesions with intermediate balloon calcification in a case of nodular pulmonary amyloidosis: A case report

**DOI:** 10.1097/MD.0000000000034964

**Published:** 2023-09-08

**Authors:** Mingquan Yin, Lecong Ouyang, Jianlong Tan, Wei Liu, Weidong Zhang

**Affiliations:** a Department of Respiratory and Critical Care Medicine, The First Affiliated Hospital of Hunan Normal University/Hunan Provincial People’s Hospital, Changsha, China.

**Keywords:** calcification, EBUS-TDB, lung cyst, pulmonary amyloidosis

## Abstract

**Rationale:**

Amyloidosis is a rare disease characterized by the misfolding of autologous proteins and extracellular deposition of fibrils, which can involve 1 or more vital organs in the body. Nodular pulmonary amyloidosis with extensive pulmonary cysts is even less common. This study discusses the diagnosis and treatment of a case of pulmonary nodular amyloidosis with extensive pulmonary cysts and calcification in the middle of the cysts on chest computed tomography, and reviews the related literature. We hope that this rare case will raise awareness of this disease among clinicians.

**Patient concerns:**

Multiple pulmonary nodules and cysts were found on computed tomography of the chest, and the patient was eager to further clarify the nature of the nodules and the next treatment plan.

**Diagnoses:**

Amyloidosis of pulmonary nodules.

**Interventions and outcomes:**

Since the patient’s primary lesion was outside the bronchial lumen near the hilum, we opted for endobronchial ultrasound-guided tunnel biopsy to obtain pathological specimens, and confirmed the diagnosis of nodular pulmonary amyloidosis. After a definite diagnosis, the patient was regularly followed without any specific treatment.

**Lessons:**

For patients with multiple pulmonary nodules combined with extensive pulmonary cysts, we also need to be alert to the possibility of pulmonary nodule amyloidosis. Secondly, when the main lesion is located outside the bronchial cavity near the hilum of the lung, the method of pathological tissue biopsy should also consider endobronchial ultrasound-guided tunnel biopsy.

## 1. Introduction

Amyloidosis is a disease associated with a variety of genetic, inflammatory and neoplastic diseases, characterized by extracellular deposition of misfolded proteins, causing tissue damage and organ failure. The disease can be limited to a single organ or involve multiple organs. In clinical practice, we often can be divided into systemic and localized according to the sites involved, and the respiratory system is the most common site involved. Here we report a rare case of nodular pulmonary amyloidosis with specificity in obtaining pathological specimens and chest imaging.

## 2. Case presentation

**Figure 1.**
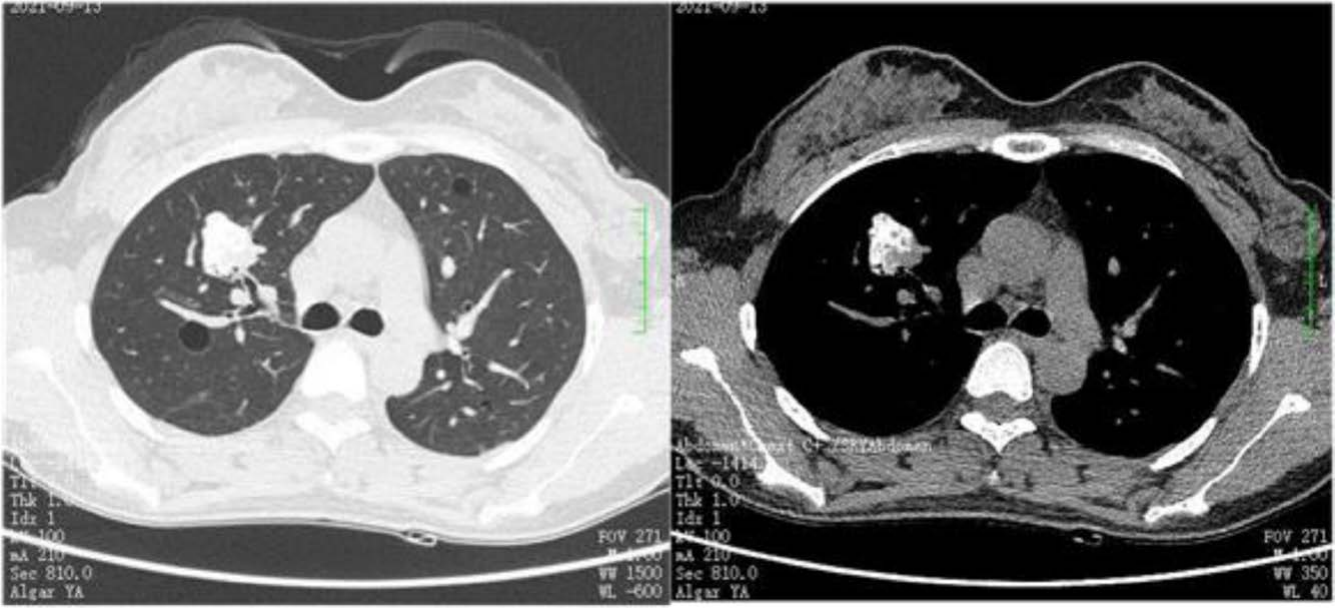
Multiple nodules were found in both lungs, and the larger one was located in the anterior segment of the right upper lung.

**Figure 2.**
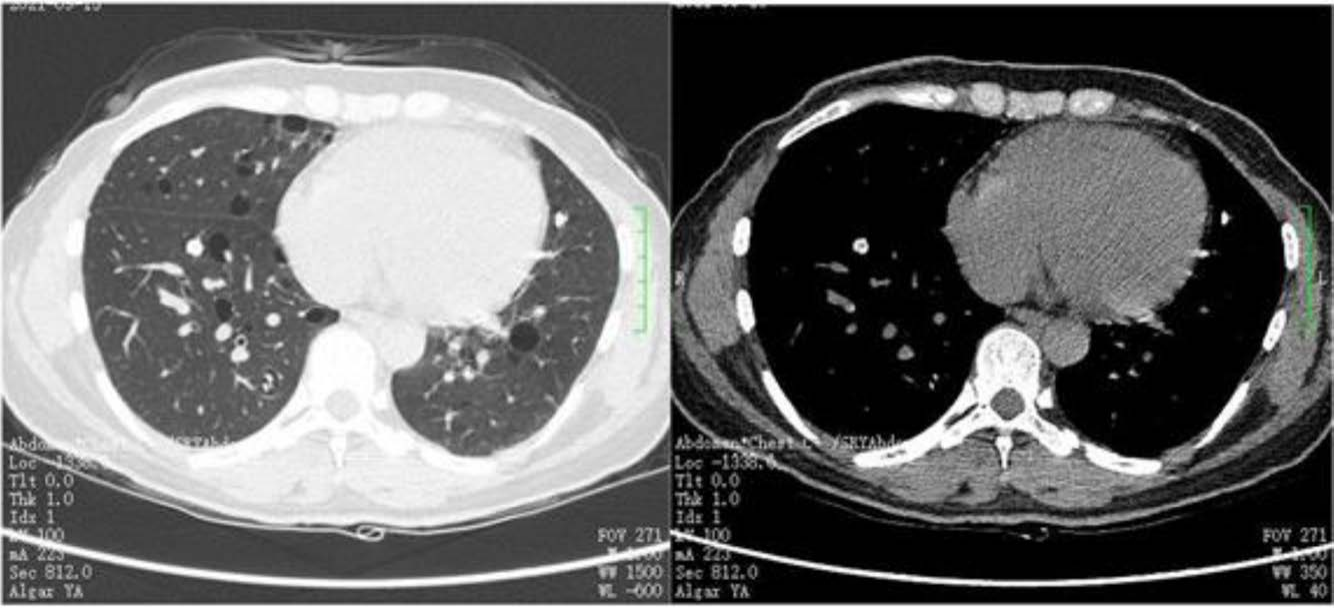
Multiple calcified lesions in both lungs.

**Figure 3.**
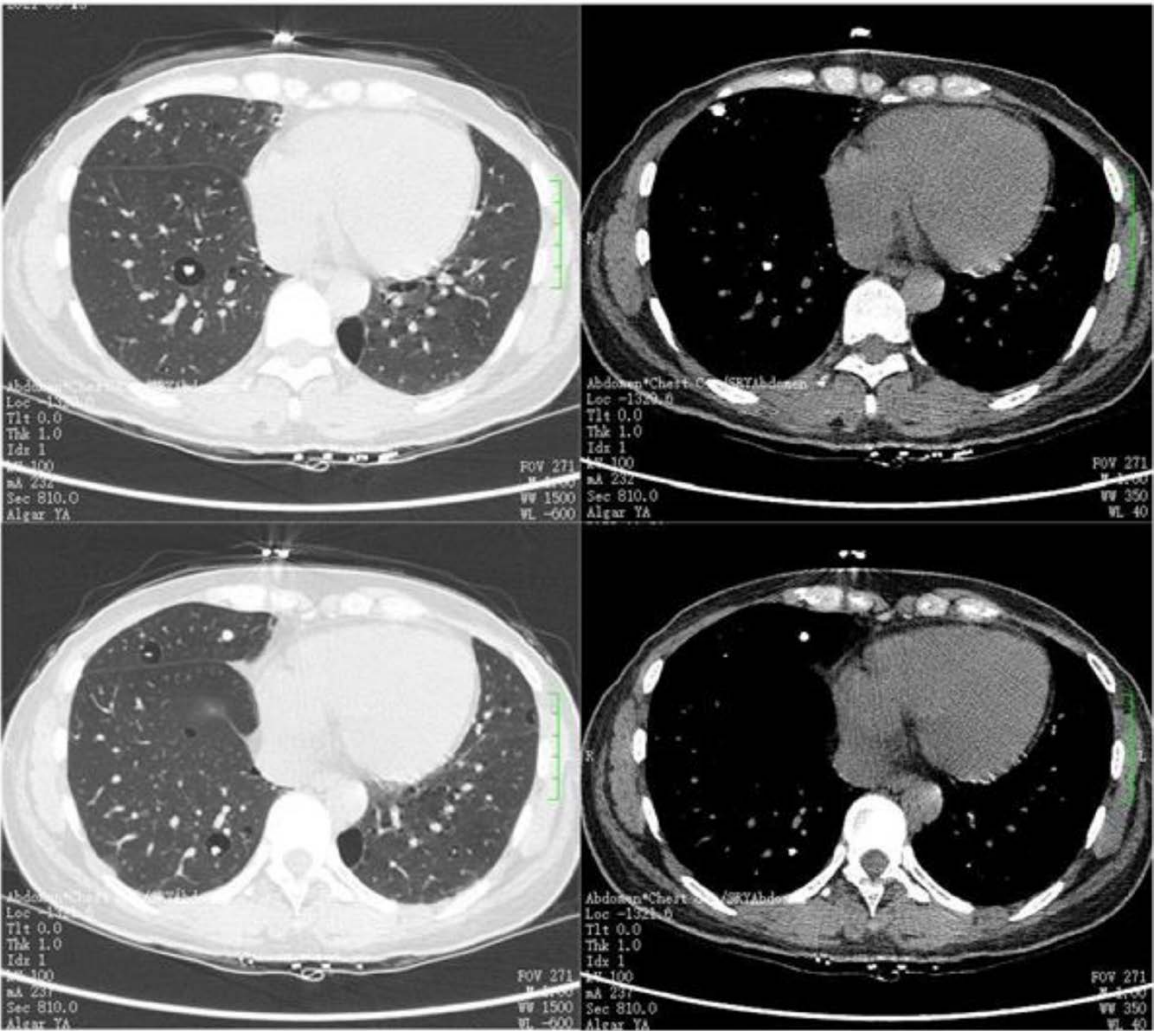
Multiple lung sacs in both lungs, apparent calcification foci in the middle of cysts.

A 41-year-old Chinese woman presented to our outpatient clinic for reexamination because pulmonary nodules were found accidentally on a routine chest radiograph for half a year. She has no history of smoking, drug or food allergy, chronic respiratory disease, autoimmune disease, or exposure to epidemic water. The patient denied clinical manifestations of cough, expectoration, fever, hemoptysis, dyspnea, reduced endurance, or recent weight loss, no family history of lung cancer. On physical examination, breath sounds were evident in both lungs without rales. Enlarged cervical, supraclavicular, or axillary lymph nodes were not touched. Chest enhanced computed tomography (CT) showed that: Both lungs mainly diverged in nodules, and the larger 1 was located in the anterior segment of the right upper lung, about 23 mm × 20 mm in size, with uneven internal density and multiple calcification foci (Fig. 1); Multiple calcified lesions in both lungs (Fig. 2); Multiple lung sacs in both lungs, apparent calcification foci in the middle of cysts (Fig. 3); No pleural effusion was found. No enlarged lymph nodes were observed in the mediastinum. Multiple lesions in both lungs and the diagnosis of amyloidosis pending exclusion.

To further clarify the diagnosis, the patient underwent a transbronchial lung biopsy, which resulted in a definitive diagnosis. The location of the amyloid lesion was outside the bronchial lumen, which increased the difficulty in obtaining pathological tissue. For percutaneous lung biopsy or transtracheal ultrasound biopsy, we finally chose the latter after discussion. After obtaining the patient’s consent, we finally performed endobronchial ultrasound-guided tunnel biopsy (EBUS-TDB), successfully punctured the RB3a branch of the patient’s right lung to establish a tunnel, and obtained effective pathological tissue through tunnel forgings.

**Figure 4.**
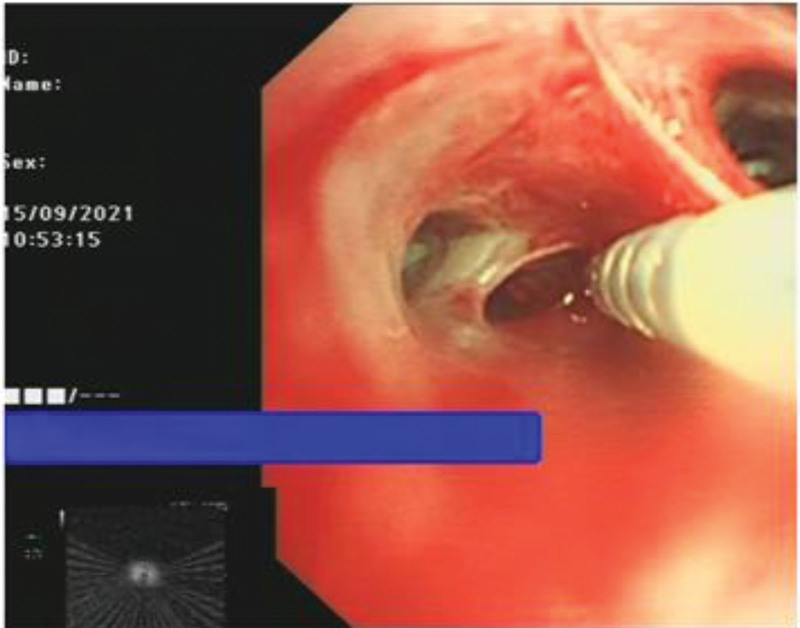
Abnormal ultrasound images could be detected at RB3a, and Wang wire was used for repeated transmural puncture three times.

**Figure 5.**
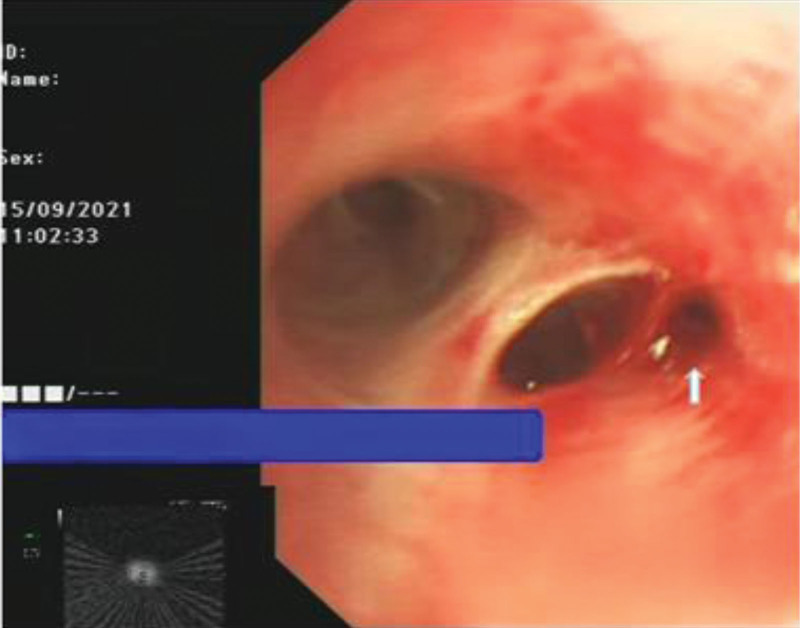
Tunnels formed after repeated Wang needle punctures at the same site (Where the white arrow points).

**Figure 6.**
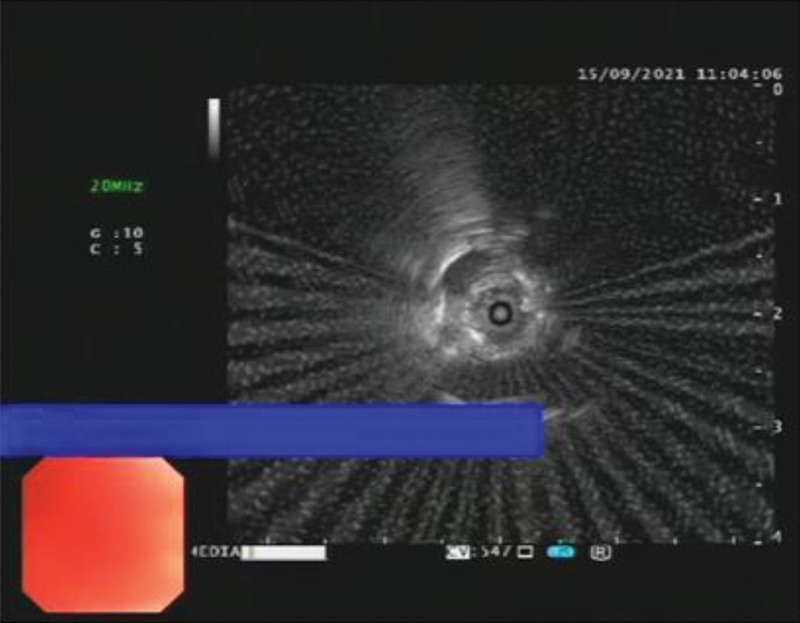
Through the tunnel formed after the puncture, endoscopic ultrasonography (EUS) was inserted again to detect abnormal echo.

EBUS-TDB: Under local anesthesia, the fiberoptic bronchoscope was slowly inserted into the airway, and the tracheal mucosa was smooth and intact, the lumen was patulous, and the carina was sharp. The left and right bronchial mucosa of grades 1 to 4 were smooth and intact, without stenosis, hemorrhage and new organisms. Combined with chest CT, abnormal acoustic images could be detected by endobronchial ultrasound exploration in the RB3a subbranch of the patient’s right lung. Transmural biopsy with Wang wire was performed 3 times (Fig. 4), and a tunnel was observed after a repeated puncture at the same site (Fig. 5), and the abnormal echo was detected by reinsertion of endoscopic ultrasound (Fig. 6). Biopsy forceps were used to clamp the pathological tissue through the tunnel and send it to biopsy. The patient’s condition was stable after the operation, with no obvious bleeding or pneumothorax.

**Figure 7.**
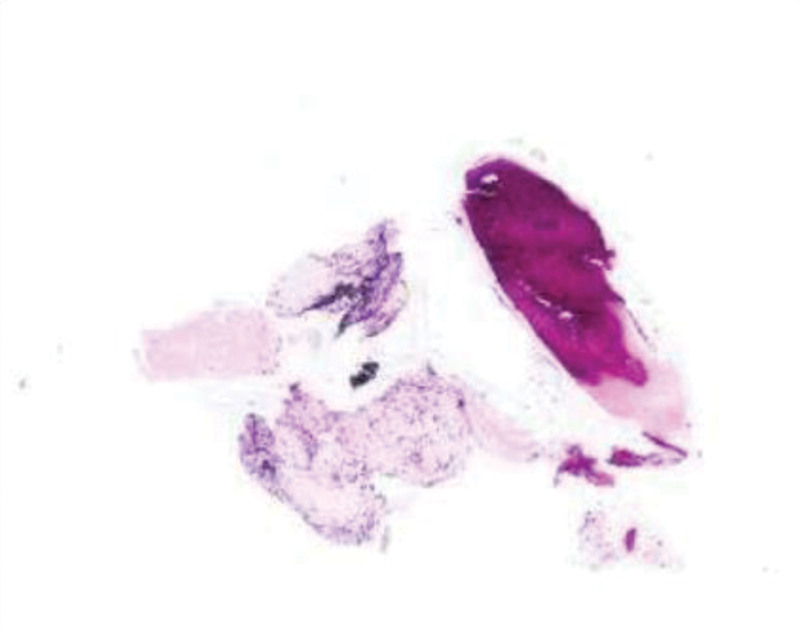
(Tissue of right upper lobe): Special stain: Congo Red (+).

Pathological Tissue Biopsy showed that: (Tissue of right upper lobe) amyloidosis in some connective tissue, calcification and ossification in others. Special stain: Congo Red (+) (Fig. 7).

In terms of treatment, because the patient had no obvious clinical symptoms and there was no sufficient evidence of systemic amyloidosis, we decided to follow up and observe the patient regularly.

## 3. Discussion

Amyloidosis is a rare disease characterized by the misfolding of autologous proteins and extracellular deposition of fibrils, which can involve 1 or more vital organs in the body. Amyloidosis can be classified as systemic or localized according to the site of involvement. The 4 common subtypes of systemic amyloidosis are secondary amyloid A amyloidosis, primary light-chain amyloidosis, β2-microglobulin associated amyloidosis, and familial amyloidosis.^[1]^ Pulmonary amyloidosis may be part of localized or systemic amyloidosis. There are 3 main tissue manifestations of pulmonary amyloidosis: diffuse alveolar septal amyloidosis, tracheobronchial amyloidosis, and nodular pulmonary amyloidosis.^[2]^ In 1983, a review of the literature on localized amyloid lung disease counted that of the 126 cases found, 44% had nodular disease, 3% had diffuse interalveolar amyloidosis, and 53% had tracheobronchial amyloid.^[3]^

Diffuse alveolar septal amyloidosis is the rarest form of pulmonary amyloidosis and usually occurs in patients with systemic amyloidosis. The deposited amyloid causes damage to the alveolar walls and capillaries, leading to impaired gas exchange. Patients usually present with cough, hemoptysis, dyspnea and even respiratory failure; This type usually has a poor prognosis.^[1,4]^ Imaging features often include thickening of the interlobular septa, consolidation of the subpleural area, and ground-glass infiltration.^[5]^

Tracheobronchial amyloidosis usually manifests as associated pulmonary symptoms secondary to airway stenoses, such as dyspnea, cough, recurrent pneumonia, or atelectasis.^[4]^ Irregular lumen, thickened bronchial wall and calcified amyloid deposition are typical radiological features of tracheobronchial amyloidosis.^[5]^ There is no specific drug treatment for tracheobronchial amyloidosis. Radiation therapy and/or bronchoscopic intervention are usually chosen.^[6]^

Nodular pulmonary amyloidosis is usually asymptomatic at the diagnosis (as in our case). Its lesion is usually local, conservative resection is usually curative, and the long-term prognosis is the best of the 3 types.^[6]^ Various studies reported many experts believe that the pathogenesis of most nodular pulmonary amyloidosis is related to lymphoproliferative diseases or chronic immune inflammation, such as mucosa-associated lymphoid tissue (MALT) exordial marginal zone lymphoma, systemic lupus erythematosus (SLE) or Sjogren’s syndrome (SS). Jacob M et al reported a case of nodular pulmonary amyloidosis associated with latent MALT lymphoma in a patient with known SLE.^[4]^ Yoshiaki K et al reported a case with directed radiological features of nodular pulmonary amyloidosis associated with SS.^[7]^ Hua Xiang et al reported a case of nodular pulmonary amyloidosis with extensive plasma cell differentiation and marked ossification caused by primary lung MALT lymphoma.^[2]^

Nodular pulmonary amyloidosis often appears on CT images as pulmonary nodules located subpleural or peripheral and in the lower lobe. Pulmonary nodules have sharp lobulated edges. Nodules vary in size and shape and may be single or multiple.^[8]^ Over time, nodules may grow slowly, calcify, balloon-like changes, or even break down spontaneously.^[9]^ The mechanism by which amyloidosis forms cysts is still unclear. However, there are 3 possible mechanisms leading to cyst formation: Ohdama et al^[10]^ speculated that it may be relevant to a massive inflammatory cell infiltration, and airway stenosis leads to the ball valve mechanism. Amyloid deposition on the alveolar wall increases the fragility and destruction of the alveolar wall may be another potential mechanism.^[11]^ Furthermore, the deposition of amyloid proteins in pericapillary and interstitial tissues, which clog the alveolar capillaries, leading to ischemia and destruction of the alveolar wall may be also contributed to the formation of nodular.^[9]^

Although medical reports of pulmonary amyloidosis are not uncommon, our present report is unusual in that it is a case of nodular pulmonary amyloidosis with multiple balloon-like lesions in the lung on CT with intermediate balloon calcification. In contrast to the reported cases, our patient had no clinical signs or symptoms of SS or SLE, and the bronchial biopsy specimen obtained this time did not show evidence of lymphatic hyperplasia. In addition, Chew KM et al reported a case of nodular pulmonary amyloidosis with extensive pulmonary cysts in both lungs without any underlying inflammatory or proliferative disease.^[9]^ Compared with the case reported by Chew KM et al, in our case, not only an unusual phenomenon of multiple pulmonary cysts far from the calcification focus was observed on the lung CT image, but also obvious calcification was observed in the middle of the air sac of the right lower lung, which was a rarer CT manifestation. The pathogenesis of calcification in the middle of the right lower lung balloon is still unclear. We hypothesized that: Possibly due to a large inflammatory cell infiltration, the bronchi become edematous and constricted, squeezing the alveolar capillaries, resulting in ischemia, necrosis, and sac-like degeneration of the alveoli, while the middle, already constricted bronchus itself develops amyloid deposition and calcification over time.

In addition, another characteristic of our case is the specificity of the method used to obtain the pathological tissue specimen for examination. As far as I know, most other investigators have obtained tissue specimens by CT-guided percutaneous lung biopsy^[1,5,9]^, or lung wedge resection to further confirm the diagnosis.^[2–4]^ But in our case, the pulmonary nodule was significantly calcified and no pathological specimen could be obtained by percutaneous puncture. Combined with the patient’s chest CT, we preliminarily determined that the significant lesion was located in the right lung near the hilar, outside the bronchial lumen. Therefore, with the patient’s consent, we chosen EBUS-TDB, and successfully obtained pathological tissue.

EBUS-TDB is an interventional respiratory technique that under the guidance of endobronchial ultrasound, a tunnel is established through the mucosa and submucosal tissue of the main airway, and then biopsy samples are obtained from the mediastinal lesions or lesions adjacent to the main airway. This technique includes 3 steps: endobronchial ultrasound-guided transbronchial needle aspiration (EBUS-TBNA), tunneling and biopsy forceps. EBUS-TDB is an extension and development of endobronchial ultrasound-guided transbronchial node biopsy.^[12]^ Compared with percutaneous lung puncture or pneumonectomy, EBUS-TDB is a safer technique.^[13]^ Besides, a study by Bramley K et al^[14]^ shows that the sensitivity and specificity of EBUS-TDB is better than EBUS-TBNA in the diagnosis of benign lesions. And just like our case, not only did we obtain a sufficient amount of pathological samples by this method, but the patient also had no postoperative hemorrhage or pneumothorax. Therefore, EBUS-TDB is also a good method for the diagnosis of this benign lung disease. We strongly advocate this technique as an alternative to EBUS-TBNA for thoracic surgeons and advanced bronchoscopists in select clinical scenarios.

## 4. Conclusion

We report a rare case of nodular pulmonary amyloidosis in which pathological tissue was obtained using a special method EBUS-TDB. Moreover, our case was associated with multiple lung sacs with calcification in the center of the sacs, which is also a rare manifestation in pulmonary nodular amyloidosis.

## Acknowledgements

The authors would like to thank the investigators and patients at the investigative sites for their support of this study.

## Author contributions

**Conceptualization:** Jianlong Tan, Wei Liu.

**Data curation:** Mingquan Yin, Lecong Ouyang.

**Funding acquisition:** Mingquan Yin, Jianlong Tan, Weidong Zhang.

**Project administration:** Weidong Zhang.

**Resources:** Weidong Zhang.

**Validation:** Lecong Ouyang.

**Visualization:** Wei Liu, Weidong Zhang.

**Writing – original draft:** Mingquan Yin.

**Writing – review & editing:** Mingquan Yin, Jianlong Tan, Wei Liu, Weidong Zhang.
